# *LMX1B* Locus Associated with Low-Risk Baseline Glaucomatous Features in the POAAGG Study

**DOI:** 10.3390/genes12081252

**Published:** 2021-08-16

**Authors:** Elana Meer, Vivian L. Qin, Harini V. Gudiseva, Brendan McGeehan, Rebecca Salowe, Maxwell Pistilli, Jie He, Ebenezer Daniel, Gui Shang Ying, Venkata R. M. Chavali, Joan M. O’Brien

**Affiliations:** 1 Perelman School of Medicine, University of Pennsylvania, Philadelphia, PA 19104, USA; Elana.Meer@pennmedicine.upenn.edu; 2 Scheie Eye Institute, University of Pennsylvania, Philadelphia, PA 19104, USA; Vivian.Qin@pennmedicine.upenn.edu (V.L.Q.); gudiseva@pennmedicine.upenn.edu (H.V.G.); brenmc@pennmedicine.upenn.edu (B.M.); Rebecca.Salowe@pennmedicine.upenn.edu (R.S.); pistilli@gmail.com (M.P.); jhe2@pennmedicine.upenn.edu (J.H.); ebdaniel@pennmedicine.upenn.edu (E.D.); gsying@pennmedicine.upenn.edu (G.S.Y.); vchavali@pennmedicine.upenn.edu (V.R.M.C.)

**Keywords:** primary open-angle glaucoma, glaucoma, *LMX1B*, genetic, African American

## Abstract

Primary open-angle glaucoma (POAG) is the leading cause of irreversible blindness worldwide and has been associated with multiple genetic risk factors. The *LMX1B* gene is a genetic susceptibility factor for POAG, and several single-nucleotide polymorphisms (SNPs) were shown to be associated with POAG in our own prior Primary Open-Angle African American Glaucoma Genetics (POAAGG) study genome-wide association study (GWAS). This study evaluated the association of the *LMX1B* locus with baseline optic disc and clinical phenotypic characteristics of glaucoma patients from our African American cohort. Compared to the GG genotype in SNP rs187699205, the GC genotype in this SNP was found to be significantly associated with a smaller cup-to-disc ratio (CDR) and increased (better) visual field mean deviation (MD) in glaucoma cases. None of the glaucoma cases with the GC genotype had disc hemorrhages, disc notching, or beanpot disc appearance. In conclusion, glaucoma phenotypes differed significantly by *LMX1B* variant in African American patients with POAG, and a SNP variant was associated with certain disease features considered lower risk.

## 1. Introduction

Primary open-angle glaucoma (POAG) is a complex neurodegenerative disease characterized by optic nerve atrophy and resultant progressive loss of vision [1,2]. POAG has been associated with multiple environmental and genetic risk factors, including elevated intraocular pressure (IOP), advanced age, family history, and African ancestry [3,4]. In recent years, family linkage and genome-wide association studies (GWAS) have demonstrated the importance of genetics in the development and progression of glaucoma [4,5,6]. Population-based studies have demonstrated significantly elevated risk and prevalence of glaucoma in first-degree relatives of patients [7] and twin studies have shown a high degree of concordance among monozygotic twins [8]. Furthermore, recent GWAS have implicated multiple genes and genetic loci associated with susceptibility to POAG [5].

The *LMX1B* gene is one such example of a susceptibility factor for the development of POAG [9,10]. *Lmx1b* in mice encodes a LIM homeobox transcription factor responsible for regulating gene expression required for normal morphogenesis and cell differentiation of the anterior segment, including specifically the trabecular meshwork (TM) and ciliary body, which are responsible for aqueous drainage and production [11]. In humans, *LMX1B* mutations cause Nail-Patella Syndrome (NPS), an autosomal dominant condition of the nails and patellae, in which around 33% of patients over 40 years old develop open-angle glaucoma (compared to 1–2% of the general population over 40 years old) [12]. Recent GWAS studies demonstrated that *LMX1B* variants are associated with elevated IOP and POAG, even without a diagnosis of NPS or extraocular involvement, and in the absence of anterior segment abnormalities [9]. Interestingly, in our Primary Open-Angle African American Glaucoma Genetics (POAAGG) GWAS study, the variants in *LMX1B* were significantly associated with mean deviation (MD) baseline, but not with IOP [13]. There have been relatively few studies specifically investigating the genetic risk factors for glaucoma in African American populations, even though this population has a higher prevalence of POAG and more severe outcomes [14,15].

In this study, we further refined the association of the *LMX1B* locus with POAG in the POAAGG cohort, and established whether mutations in this region are associated with specific phenotypic characteristics corresponding to severity and progression of disease.

## 2. Materials and Methods

### 2.1. Study Subject Recruitment

The POAAGG study population includes self-identified African Americans (Black, Afro-Caribbean, or African descent), aged 35 years or older. Subjects were recruited from all comprehensive and subspecialty ophthalmology clinics at the University of Pennsylvania and at external ophthalmology clinics in Philadelphia (Windell Murphy, MD; Temple University). The baseline demographics and the inclusion and exclusion criteria of the POAAGG study have been described previously [16]. All subjects received a clinical examination, including an onsite interview and ophthalmic examination. Examination data were recorded on case report forms, which were entered into the REDCap (Research Electronic Data Capture) database. Additionally, we extracted retrospective ophthalmic and systemic health data from the UPenn EPIC and MERGE databases.

Fellowship-trained glaucoma specialists classified subjects as glaucoma cases, glaucoma suspects, or controls. Glaucoma cases were defined as having an open iridocorneal angle and: (1) at least one characteristic glaucomatous optic nerve finding (notching, neuroretinal rim thinning, excavation, or a nerve fiber layer defect); (2) characteristic visual field defects on two consecutive reliable visual field tests in at least one eye, which were consistent with the observed optic nerve defects in that eye; and (3) no identified secondary cause of glaucoma. Normal controls were defined as subjects seen in scheduled ophthalmology appointments, without a glaucoma diagnosis or confounding ocular conditions. Informed consent was obtained from all participating subjects. Research was conducted with the approval of the University of Pennsylvania Institutional Review Board and in accordance with the Declaration of Helsinki.

### 2.2. Genotyping

The genotypes of the *LMX1B* gene were generated by using Illumina MEGA array chip. Data generation, quality control, and single variant associations’ tests were performed as described in the POAAGG GWAS [13]. In brief, the genotype calls were generated using the Genome Studio genotyping module (GT). After the quality control and single variant associations analysis, *LMX1B* genotypes were extracted from the data. These genotypes were further used in this study.

### 2.3. Deep Phenotyping of Cases

Phenotypic data for POAG cases were extracted from the REDCap Database, and glaucoma cases were grouped for analysis by *LMX1B* SNP genotype (e.g., GG and GC), which have previously been described by our group [13]. Genotype was masked during chart review for data extraction. Comparisons of phenotypic data between the GG and GC variants were limited to the glaucoma case group only, as most controls had limited phenotypic data due to their lack of glaucoma diagnosis. A total of 2570 cases were included in the downstream analysis with quantitative and qualitative glaucoma phenotype features.

The following baseline phenotypic information was collected at the time of enrollment for each subject: visual acuity (VA), mean IOP, central corneal thickness (CCT), cup-to-disc ratio (CDR), retinal nerve fiber layer thickness (RNFL) on optical coherence tomography (OCT), and MD and pattern standard deviation (PSD) on Humphrey visual field 24-2 test.

Qualitative optic nerve characteristics were obtained using a standardized grading form for baseline 30-degree color stereo disc photos of POAAGG subjects, which were taken using the Topcon TRC 50EX retinal camera (Topcon Corp. of America, Paramus, NJ, USA). Two non-physician graders, trained by glaucoma specialists [17], independently completed this grading form for each image, assessing a number of features of the optic cup and disc, masked to the genotype data. Subjects were excluded if they did not have disc photos or if the disc photos were deemed too poor quality to proceed with grading. The following data were collected from the graders’ forms: disc/cup characteristics, peripapillary atrophy characteristics, and features of glaucomatous optic nerves. 

### 2.4. Statistical Analysis

A total of 6 SNPs from the *LMX1B* region which are in linkage disequilibrium (LD) were found to be associated with MD in our earlier study [13]. The 6 linked SNP variants identified were rs143217136, rs115683895, rs144229999, rs147720587, rs140140891, and rs187699205. To our knowledge, none of these *LMX1B* SNPs have been previously described in the literature. As these 6 SNPs segregate together as a haplotype, rs187699205 was chosen for analysis as a representative SNP. 

Measurements closest to the study enrollment date were chosen as the baseline measures for statistical analysis. For the continuous clinical phenotype measurements, the descriptive means and standard deviations (SD) by genotype groups were reported. For categorical phenotype measurements, the counts and percentages were reported. Comparisons of the genotype distributions between POAAGG cases and controls were performed using Fisher’s exact tests, due to small cell counts in one or more cells. For analysis of the SNP rs187699205 among glaucoma cases, only one subject had the CC genotype; this subject was excluded from the statistical comparisons of phenotype measurements for SNP rs187699205 genotypes (e.g., GG vs. GC). Comparisons of the baseline ocular clinical phenotypes and optic disc parameters between the genotype groups (e.g., GG vs. GC) were performed using generalized estimating equations (GEE) to account for inter-eye correlation. From the GEE models, the model-estimated mean difference, 95% confidence interval (95% CI), and *p*-value were obtained for continuous measurements. All analyses were performed in R version 4.03. 

## 3. Results

### 3.1. Distribution of LMX1B Variants in Study Population

A total of 5825 subjects were included in the present study. Of these, 3254 (55.9%) were controls and 2571 (44.1%) were POAG cases. A total of 5690 (97.7%) subjects (3180 controls, 2510 cases) harbored a GG genotype, while 130 (2.2%) subjects (70 controls, 60 cases) harbored a GC genotype. Only 5 (0.1%) subjects (4 controls, 1 case) harbored a CC genotype (Table 1). The distribution of *LMX1B* genotypes between cases and controls was not statistically significant (Table 1). The distribution of *LMX1B* genotypes in linkage disequilibrium with the SNP rs187699205 also did not reach statistical significance (Table 2).

### 3.2. Phenotypic Characteristics of Cases for LMX1B Variant rs187699205

When compared to the GG cases, GC cases demonstrated a smaller CDR (mean difference between groups −0.07, *p* = 0.003) and an increased (less severe) visual field MD (mean difference 3.54, *p* < 0.001) (Table 3). All other phenotypic characteristics did not demonstrate a statistically significant difference between the GG and GC groups. 

### 3.3. Optic Disc Parameters of Cases for LMX1B Variant rs187699205

There was no significant difference between the GG and GC variant groups with regard to disc shape (round or oval) (Table 4). There were more eyes in the GG group with an optic disc size that were graded ‘abnormal’ (i.e., graded as a microdisc or macrodisc) than in the GC group (Table 4). 

There was a significant difference in the shape of optic cups between the GG and GC variant groups (*p* < 0.001). In the GC group, 12 (31.6%) had a conical cup shape, 26 (68.4%) had a cylindrical shape, and 0% had a beanpot shape. In the GG group, 711 (36.2%) had a conical shape, 998 (50.8%) had a cylindrical shape, and 257 (13.1%) had a beanpot shape. The cup depth did not differ significantly between the variant groups, with similar numbers of shallow, moderate, and severe cup depth in both groups.

A larger percentage of eyes in the GG group demonstrated disc hemorrhage (2.1% versus 0%, *p* < 0.001) and had observed disc notching (6.0% versus 0%, *p* < 0.001), compared to eyes in the GC group (Table 4). There was no significant difference in rim position, location of rim depression, presence of peripapillary atrophy, presence of disc excavation, presence of heavy pigmentation, tilted disc, or disc pallor between the GG and GC groups.

## 4. Discussion

This study investigated the association of POAG-associated SNPs in the *LMX1B* gene with POAG in an African American cohort [13]. We compared the phenotypic characteristics and optic disc parameters of glaucoma patients between the GC and GG genotype in the SNP rs187699205 at chromosome 9:129449650 in the intron of *LMX1B* gene. We found that the GC genotype may be associated with features of decreased POAG disease severity in a cohort of African American subjects. 

*LMX1B* has previously been identified as a glaucoma susceptibility locus [9]. Murine studies have shown that *Lmx1b* mutations cause elevated IOP and glaucomatous nerve damage, both in the presence and in the absence of structural or developmental ocular abnormalities [11]. Studies in humans have also demonstrated the phenotypic variability of glaucoma in patients with Nail-Patella Syndrome, which is caused by mutations in *LMX1B* [18]. Furthermore, variants in *LMX1B* have been associated with POAG, including the identification of protective haplotypes seen less frequently in patients with glaucoma or elevated IOP [9]. A single-nucleotide polymorphism (SNP) near the *LMX1B* gene is associated with combined OAG and cup area in a genomic study of patients of European ancestry [19]. In the POAAGG GWAS study, we identified significant SNPs in the *LMX1B* gene associated with visual field mean deviation [13], which we further analyzed in the present study. 

The present study shows that glaucoma phenotypes differ significantly by *LMX1B* variant in African American patients with POAG. From baseline examinations, the GC genotype of the SNP rs187699205 was associated with a smaller CDR and increased (better) visual field MD than the GG variant. Larger CDR is known to be a predictor of POAG from the landmark Ocular Hypertension Treatment Trial [20,21], and recent genetic studies have identified variants associated with enlarged CDR that are risk factors for POAG in US Caucasian patients [22,23]. Decreased (more severe) mean deviation measured on visual field has also been reported to be associated with risk of further progression and faster rate of progression on subsequent visual field measurements [24,25,26]. Thus, these findings suggest that patients with the rs187699205 SNP may be at lower risk for rapid progression of their glaucomatous damage when compared to patients with the GG variant. 

No eyes with the GC genotype demonstrated a beanpot optic disc appearance, disc hemorrhage, or disc notching, all factors that have been associated with glaucoma [27]. A beanpot description of the optic nerve refers to a deep appearance of the optic nerve cup in which the lamina cribrosa is posteriorly displaced and the neuroretinal rim is undermined, and has been described in eyes with high myopia as well as eyes with ocular hypertension [28,29]. Disc hemorrhages and disc notching are both features that have well-established associations with glaucoma progression [30,31,32,33,34]. The absence of these characteristics in patients harboring the GC genotype further suggests a potential protective or less severe manifestation of POAG. 

Taken together, these associations suggest a possible decreased severity of glaucoma phenotype in African American patients with POAG who have the rs187699205 SNP in the *LMX1B* gene. Interestingly, there was no significant difference between the GC and GG variant groups in mean IOP. This could suggest a difference in POAG phenotype and disease progression between the subjects that is IOP-independent.

The mechanism of action and specific role of *LMX1B* in POAG has not yet been fully elucidated. Studies have shown that there is a higher expression of *LMX1B* in TM, corneal endothelium, and corneal stroma, and that expression levels may be altered by risk variants [19]. Some insight into the role of *LMX1B* may be gleaned from studies in nephrology that have shown that *Lmx1b* regulates expression of collagen genes in the kidney [35]. Given that mice lacking *Lmx1b* show defective collagen fibrillogenesis in the eye [11], it is possible that altered activity levels of *LMX1B* change the function and structure of the ocular anterior segment via changes in the collagen pathway, predisposing those eyes to the development and progression of glaucoma. Collagen helps to compose the extracellular matrix (ECM) and studies have implicated collagen in the biomechanics and treatment of glaucoma [36,37]. In our previous study, *LMX1B* was moderately expressed in all ocular tissues, was overexpressed in induced pluripotent stem cell retinal ganglion cells, and was suppressed after exposure to oxidative stress [13]. Genetic variations leading to inter-individual differences in collagen and ECM properties may alter biomechanical properties of the aqueous humor outflow pathway, and thus result in difference in susceptibility to glaucoma. 

Genetics in POAG is complex and multifactorial, and there are limitations to this study. The frequency of the GC genotype was low in the current study, which can lead to bias in the differences between groups. Further replication in larger datasets should be performed. There were optic nerve characteristics that were found to be statistically significant between the GG and GC genotype groups: disc size, nasal rim depression, and borders of peripapillary atrophy. However, given the much larger number of cases with the GG variant and the much fewer number of cases with the GC variant, these differences may not be clinically significant. Optic nerve parameters from stereoscopic images were determined by trained graders with an adjudication process; however, there is always a possibility of subjectivity in grading. Additionally, some patients did not have available or high-quality images and thus had to be excluded from the analysis, which may have introduced a degree of selection bias. 

In conclusion, this study supports the role of the *LMX1B* gene in POAG, and identifies a variant that may confer a decreased severity of disease phenotype in a cohort of African American patients. Future work needs to be done to determine the precise pathophysiology of *LMX1B* in the human eye.

## Figures and Tables

**Table 1:** Distribution of *LMX1B* genotypic variants between POAG cases and controls.

		Case/Control		
Chromosome Location rsID		Controls	Cases	Total
chr 9:129449650_C rs187699205	**GG**	3180 (97.7%)	2510 (97.6%)	5690 (97.7%)
	**GC**	70 (2.2%)	60 (2.3%)	130 (2.2%)
	**CC**	4 (0.1%)	1 (0.0%)	5 (0.1%)
	**Total**	3254 (55.9%)	2571 (44.1%)	5825

Fisher’s exact test *p*-value: 0.53.

**Table 2:** Distribution of genotypic variants between POAG cases and controls for POAG-associated *LMX1B* SNPs in LD with rs187699205.

	Disease Status			
Chromosome Location rsID	Controls (*n* = 3254)	Cases (*n* = 2571)	Total (*n* = 5825)	*p*-Value ^1^
**rs143217136**				0.34
GG	3188 (98.0%)	2510 (97.6%)	5698 (97.8%)	
GA	62 (1.9%)	60 (2.3%)	122 (2.1%)	
AA	4 (0.1%)	1 (0.0%)	5 (0.1%)	
**rs115683895**				0.29
CC	3188 (98.0%)	2509 (97.6%)	5697 (97.8%)	
CT	62 (1.9%)	61 (2.4%)	123 (2.1%)	
TT	4 (0.1%)	1 (0.0%)	5 (0.1%)	
**rs144229999**				0.32
GG	3197 (98.2%)	2515 (97.8%)	5712 (98.1%)	
GA	54 (1.7%)	55 (2.1%)	109 (1.9%)	
AA	3 (0.1%)	1 (0.0%)	4 (0.1%)	
**rs147720587**				0.48
GG	3181 (97.8%)	2509 (97.6%)	5690 (97.7%)	
GC	69 (2.1%)	61 (2.4%)	130 (2.2%)	
CC	4 (0.1%)	1 (0.0%)	5 (0.1%)	
**rs140140891**				0.53
CC	3180 (97.7%)	2510 (97.6%)	5690 (97.7%)	
TC	70 (2.2%)	60 (2.3%)	130 (2.2%)	
TT	4 (0.1%)	1 (0.0%)	5 (0.1%)	

^1^: *p* value from Fisher’s Exact Test.

**Table 3:** Clinical phenotypes by *LMX1B* SNP rs187699205 among cases.

Phenotype	GG (# Eyes)	Mean (SD)	GC (# Eyes)	Mean (SD)	Mean Difference (95% Confidence Interval) *	*p*-Value
**CCT**	4451	533.22 (39.72)	107	537.66 (38.19)	−4.45 (−14.17 to 5.27)	0.37
**CDR**	4476	0.71 (0.17)	98	0.64 (0.18)	0.07 (0.02 to 0.12)	**0.003**
**IOP**	4769	17.39 (6.12)	111	16.49 (5.28)	0.90 (−0.43 to 2.23)	0.19
**MD**	3781	−8.47 (9.05)	88	−4.93 (6.39)	−3.54 (−5.00 to −2.08)	**<0.001**
**PSD**	3784	5.22 (3.47)	88	4.59 (3.15)	0.63 (−0.02 to 1.29)	0.06
**RNFL**	3803	73.24 (15.03)	92	75.07 (15.60)	−1.83 (−5.51 to 1.85)	0.33
**VA (logMAR)**	4099	0.39 (0.85)	92	0.41 (1.00)	−0.01 (−0.23 to 0.20)	0.90

*: From GEE model: phenotype = intercept + genotype; VA = visual acuity; IOP = intraocular pressure; CCT = central corneal thickness; CDR = cup-to-disc ratio; RNFL = retinal nerve fiber layer; MD = mean deviation; PSD = pattern standard deviation.

**Table 4:** Optic disc parameters by *LMX1B* genotype among cases.

	GG (*n* = 3554 Eyes)	GC (*n* = 81 Eyes)	*p*-Value
**Disc Shape**			0.75
Round	1007 (49.1%)	18 (46.2%)	
Oval	1045 (50.9%)	21 (53.8%)	
**Disc Size**			**<0.001 ^+^**
Normal	2036 (98.7%)	40 (100.0%)	
Abnormal (micro or macro)	27 (1.3%)	0 (0.0%)	
**Cup Shape**			**<0.001 ^+^**
Conical	711 (36.2%)	12 (31.6%)	
Cylindrical	998 (50.8%)	26 (68.4%)	
Beanpot	257 (13.1%)	0 (0.0%)	
**Cup Depth**			0.71
Shallow	263 (13.3%)	5 (13.2%)	
Moderate	1265 (63.8%)	27 (71.1%)	
Severe	454 (22.9%)	6 (15.8%)	
**Constant Rim Plane Position?**			0.53
No	296 (14.9%)	4 (10.5%)	
Yes	1689 (85.1%)	34 (89.5%)	
**Inferior Rim Depression?**			0.41
No	1962 (98.8%)	37 (97.4%)	
Yes	23 (1.2%)	1 (2.6%)	
**Superior Rim Depression?**			1.00
No	1985 (100.0%)	38 (100.0%)	
Yes	0 (0.0%)	0 (0.0%)	
**Nasal Rim Depression?**			**<0.001 ^+^**
No	1954 (98.4%)	38 (100.0%)	
Yes	31 (1.6%)	0 (0.0%)	
**Temporal Rim Depression?**			0.48
No	1730 (87.2%)	35 (92.1%)	
Yes	255 (12.8%)	3 (7.9%)	
**Presence of PPA?**			1.00
No	0 (0.0%)	0 (0.0%)	
Yes	2049 (100.0%)	39 (100.0%)	
**Borders of PPA**			**<0.001 ^+^**
Indistinct	229 (11.2%)	0 (0.0%)	
Distinct	1817 (88.8%)	39 (100.0%)	
**Disc Excavation?**			0.91
No	1523 (76.5%)	28 (75.7%)	
Yes	467 (23.5%)	9 (24.3%)	
**Heavy Pigmentation**			0.16
Indistinct	1553 (75.9%)	34 (87.2%)	
Distinct	493 (24.1%)	5 (12.8%)	
**Tilted Disc?**			0.42
No	1936 (94.5%)	39 (97.5%)	
Yes	112 (5.5%)	1 (2.5%)	
**Disc Hemorrhage?**			**<0.001 ^+^**
No	2007 (97.9%)	40 (100.0%)	
Yes	43 (2.1%)	0 (0.0%)	
**Disc Notching?**			**<0.001 ^+^**
No	1899 (94.0%)	39 (100.0%)	
Yes	121 (6.0%)	0 (0.0%)	
**Disc Pallor?**			0.80
No	1985 (96.8%)	39 (97.5%)	
Yes	66 (3.2%)	1 (2.5%)	

^+^: In very large studies, statistical differences can occur between groups that are clinically trivial.

## Data Availability

All POAAGG genotype files are available from the dbGap database. National Eye Institute (NEI) Primary Open-Angle African American Glaucoma Genetics (POAAGG) Study (accession number phs001312.v1.p1: https://www.ncbi.nlm.nih.gov/projects/gap/cgi-bin/study.cgi?study_id=phs001312.v1.p1 (accessed on 1 July 2021)).
